# Effects of Motivational Interviewing on Treatment Adherence among Patients with Chronic Obstructive Pulmonary Disease: a Randomized Controlled Clinical Trial

**Published:** 2018-10

**Authors:** Hamid Naderloo, Zohre Vafadar, Alireza Eslaminejad, Abbas Ebadi

**Affiliations:** 1 Department of Critical Care Nursing, Faculty of Nursing, Baqiyatallah University of Medical Sciences, Tehran, Iran,; 2 Faculty of Nursing, Baqiyatallah University of Medical Sciences, Tehran, Iran,; 3 Chronic Respiratory Diseases Research Center, National Research Institute of Tuberculosis and Lung Diseases (NRITLD), Shahid Beheshti University of Medical Sciences, Tehran, Iran,; 4 Behavioral Sciences Research Center, Life Style Institute, Nursing Faculty, Baqiyatallah University of Medical Sciences, Tehran, Iran

**Keywords:** Treatment adherence, Motivational interviewing, Chronic obstructive pulmonary disease

## Abstract

**Background:**

Treatment non-adherence is a leading cause of rehospitalization among patients with chronic obstructive pulmonary disease. Motivational interviewing is a client-centered participatory counseling strategy which enhances motivation for change. The aim of this study was to examine the effects of motivational interviewing on treatment adherence among patients with chronic obstructive pulmonary disease.

**Materials and Methods:**

This randomized controlled clinical trial was done on 54 hospitalized patients using a two-group repeated measures design. Patients in the intervention group (n=27) received motivational interviewing and lifestyle-related educations, while their counterparts in the comparison group (n=27) solely received lifestyle-related educations. Treatment adherence was measured before, one month, and two months after the intervention.

**Results:**

At baseline, there was no significant difference between the groups regarding treatment adherence (P>0.05); however, one and two months after the intervention, between-group differences regarding treatment adherence were statistically significant (P<0.05).

**Conclusion:**

Motivational interviewing promotes treatment adherence among patients with chronic obstructive pulmonary disease.

## INTRODUCTION

Pulmonary diseases have turned into a leading cause of morbidity and mortality in the world (1). Chronic Obstructive Pulmonary Disease (COPD) is one of the most prevalent pulmonary diseases. Currently, COPD is the sixth leading cause of death and it is estimated that it becomes the third leading cause of death and the fifth most debilitating disease by 2020 (2). Since 2010, COPD-related healthcare costs have been about $2.1 trillion, $1.9 trillion of which have been related to direct costs (such as the costs of healthcare services), while $0.2 trillion have been related to indirect costs (such as loss of employment). It is estimated that indirect COPD-related costs reach $4.8 trillion by 2030 (3). About 51% of COPD-related costs are related to the acute exacerbation of the disease (4). Thus, the best way for cutting such costs is to prevent disease exacerbation or recurrence. Studies showed that most COPD-related rehospitalizations can be prevented through close adherence to treatment strategies such as smoking cessation, medication therapy, clear air breathing, avoidance from going outdoor in polluted days (5), pursed-lip breathing, and lifestyle modifications (6,7).

Poor treatment adherence, particularly among patients with chronic health conditions, is one of the main health-related concerns worldwide. It is a major reason behind the failure of treatments, increased risk for disease recurrence and exacerbation, treatment prolongation, and increased healthcare costs. Moreover, the prognosis of a chronic health condition largely depends on treatment adherence (8).

Treatment adherence among patients with COPD widely varies from 22 to 78% (9). About 200 factors have been identified to contribute to treatment non-adherence (10), the most important of which are the types and the courses of the underlying diseases and their treatments, health-related knowledge, beliefs and faith in treatments, healthcare provider-client relationship, normalization of medication use (11), personality character (12), and the process of medical referrals and visitations (13).

There are different strategies, theories, and models for promoting treatment adherence, each of which focuses on certain psychological aspects of health and behavior. However, for patients with chronic conditions such as COPD, promoting treatment adherence needs strategies which actively involve patients in the process of treatment, motivate them for treatment adherence, and help them internalize healthy behaviors. Compared with other strategies, Motivational Interviewing (MI) embodies more of these attributes (14).

Motivational Interviewing was first introduced by Miller in 1983 as a primary treatment to enhance motivation for subsequent treatments. It is a client-centered participatory counseling strategy which motivates people for change. It comprises empathy and internal conflict externalization and enhances intrinsic motivation through counseling techniques such as asking open-ended questions, reflective listening, summarizing, preparation for change (15). Clinical trials on MI reported its effectiveness in facilitating weight loss among overweight people (16), modifying lifestyle among hypertensive patients (17), improving oral self-care (18), and promoting treatment adherence among patients with transient ischemic attack (19), cystic fibrosis (20), psychosis, pathological gambling, and human immunodeficiency virus infection (21).

To the best of our knowledge, no study has yet investigated the effectiveness of MI in promoting treatment adherence among patients with COPD. Thus, this study was undertaken to examine the effects of MI on treatment adherence in this patient population.

## MATERIALS AND METHODS

### Study design

This randomized controlled clinical trial was done from January to October 2015 using a two-group repeated-measure design. The trial was registered in the Iranian Registry of Clinical Trials under the registration number of IRCT201604128650N7.

Study population was comprised of all patients with COPD who lived in Tehran, Iran, and were hospitalized in Masih-Daneshvari Hospital, Tehran, Iran. During the course of the study, 200 patients were hospitalized in the study setting, 140 of them were either ineligible for the study or unwilling to participate. Thus, the remaining 60 patients were randomly allocated to a comparison and an intervention group using the block randomization method. The number of patients in each block was four. Eligibility criteria were a positive diagnosis of COPD by a pulmonologist, an age of less than 65, no comorbid serious health condition (such as stroke or diabetes mellitus), the ability to speak and understand Persian, no history of mental disorder or Alzheimer’s disease, and basic literacy skills.

### Sample size calculation

Sample size was calculated using Altman’s nomogram, a power of 0.9, a significance level of 0.05, and the standard deviation values reported by Karimi Moonaghi et al (22). Accordingly, Altman’s monogram revealed that 27 patients were needed for each group.

### Measures

Data collection instruments were a demographic questionnaire and the Adherence among Patients with Chronic Disease (APCD) questionnaire. The items of the demographic questionnaire were age, gender, marital and educational status, insurance coverage, satisfaction with personal financial status, cigarette smoking status (pack/year), duration of affliction by COPD, drug history, disease severity base on The Global Initiative for Chronic Obstructive Lung Disease (GOLD)(2), place of residence, and history of hospitalization in the last year. APCD was developed .This questionnaire contains forty items in seven dimensions, namely making effort for treatment (9 items), intention to take treatment (7 items), adaptability (7 items), integrating illness into life (5 items), sticking to treatment (4 items), commitment to treatment (5 items), and indecisiveness for using treatments (3 items). The items are scored using a six-point Likert scale from 0 (stands for “Never”) to 5 (stands for “Completely”), resulting in a total score of 0–200. Higher scores represent closer treatment adherence. The Cronbach’s alpha and the test-retest correlation coefficient of the Persian APCD were 0.921 and 0.875, respectively (23).

### Intervention

Initially, patients in both groups completed the study questionnaires. Then, patients in the comparison group attended two training sessions on lifestyle, respiratory chest physiotherapy, and medication use. Both sessions were held in one day and lasted 15–45 minutes depending on participants’ needs and tolerance. Patients in the intervention group were provided with five one-to-one MI sessions. These sessions were held in two consecutive days. The first session was held to introduce MI and prepare patients for it. In the second session, we focused on patients’ feelings in order to help them move from extrinsic toward intrinsic motivation for change. The third session dealt mainly with identifying and resolving patients’ ambivalences and uncertainties. The aim of the fourth session was to create and stimulate an intrinsic desire for change as well as to identify, clarify, and acknowledge participants’ values. Finally, the main focus of the fifth session was on identifying tempting situations and closing the program. After MI sessions, patients were also provided with two sessions on lifestyle, respiratory chest physiotherapy, and medication use. The contents of these two sessions were the same for both groups and had been approved by a pulmonologist. All sessions for patients in both groups were held by the first author. The length of all sessions held for the patients in the intervention group varied from 15 to 45 minutes depending on participants’ needs and tolerance. One and two months after the last session, patients in both groups recompleted APCD questionnaire. During this two-month period, we made several telephone contacts with patients in both groups in order to answer their probable questions. It is noteworthy that the contents of the MI sessions were developed based on the Miller’s recommendations(24) and was approved by an MI specialist.

### Data analysis

Data analysis was done using the SPSS software (version 22). The Kolmogorov-Smirnov test was done to compare the study variables with the normal distribution. Moreover, the Chi-square and the independent-sample t tests were used to compare the groups regarding participants’ demographic characteristics and treatment adherence. The repeated-measure analysis of variance was also employed to compare the groups regarding the variations of treatment adherence scores across the three measurement time points.

## RESULTS

**Figure 1.**
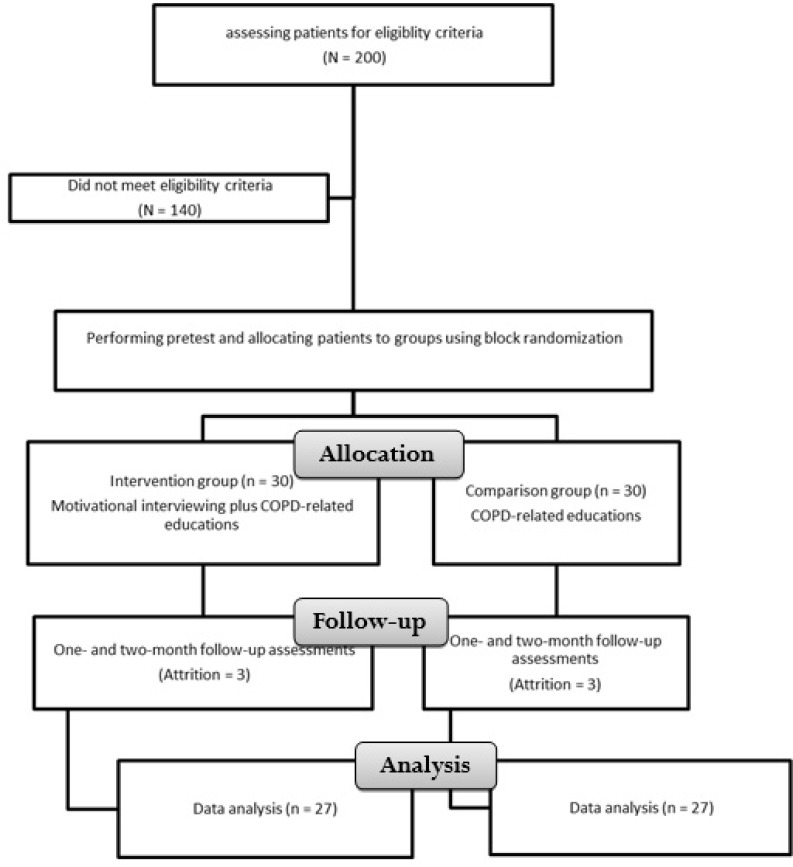
The flow of participants in the trial

In total, sixty patients were recruited to the study. In the comparison group, two patients were unwilling to stay in the study and one died during the study. Moreover, in the intervention group, three patients were unwilling to stay in the study. All these six patients were excluded and therefore, final data analysis was done on the data retrieved from 27 patients in each group. Figure 1 shows the flow of participants in the trial.

Most patients were male (57.4%) and married (79.62%) and suffered from very severe COPD (51.58%). Moreover, their age mean was about 54 and they were suffering from COPD for about nine years on average. The results of the independent-sample t, the Chi-square, and the Fisher exact tests showed no significant within-group differences regarding participants’ demographic characteristics, clinical characteristics, and drug history (P<0.05; Table 1).

**Table 1.** Comparing the groups regarding participants’ characteristics

**Group** **Characteristics**		**Comparison (Mean ± SD)**	**Intervention (Mean ± SD)**	**The results of the Independent-sample t test**
Age (year)		55.04 (7.8)	53.07 (10.06)	t = 0.76 P =0.44
Duration of affliction by COPD (year)		9.52 (9.4)	9.07 (10.6)	t = 0.16 P =0.87
Number of rehospitalizations		1.93 (1.1)	2.37 (1.4)	t =−1.2 P =0.22
Cigarette smoking (pack/year)		15.14 (23)	10.5 (15.6)	t =0.86 P =0.39
		**N (%)**	**N (%)**	**The results of the Chi-square test**
Gender	Male	17 (63)	14 (51.9)	χ^2^= 0.68 P = 0.58
	Female	10 (37)	13 (48.1)	
Marital status	Single	5 (18.5)	6 (21.2)	χ^2^= 0.114 P < 0.001
	Married	22 (81.5)	21 (77.8)	
Educational status	Elementary and less	17 (63)	9 (33.3)	χ^2^= 4.98 P = 0.083
	Under the diploma	6 (22.2)	9 (33.3)	
	Diploma and higher	4 (14.8)	9 (33.3)	
Disease severity	Mild	4 (14.8)	2 (7.4)	χ^2^= 0.81 P = 0.84
	Moderate	2 (7.4)	2 (7.4)	
	Severe	8 (29.6)	8 (29.8)	
	Very severe	13 (48.1)	15 (55.6)	

Table 2 shows the mean scores of treatment adherence and all its dimensions at different measurement time points. The results of the independent-sample t test illustrated no significant difference between the groups regarding baseline scores of treatment adherence (P>0.05).

**Table 2.** Comparing the groups regarding the mean scores of treatment adherence and all its dimensions

Treatment adherence and its dimensions	Time	Pretest (Mean ± SD)	Posttest 1 (Mean ± SD)	Posttest 2 (Mean ± SD)	Within-group repeated-measure analysis of variance
	Group				
Making effort for treatment	Comparison	34.93 (4.5)	32.3(5.3)	31.7(6)	F = 11.41
	Intervention	32.7(5.5)	36.78(4.7)	36.5(6.8)	P <0.001
	Independent-sample	t =1.59	t =−3.27	t =−.2.73	
	t test	P =0.117	P =0.002	P =0.008	
Intention to take treatment	Comparison	29 (3.6)	24.8(4.6)	23.9(4.6)	F = 15.28
	Intervention	26.7(4.8)	29(5)	28.5(5.4)	P <0.001
	Independent-sample	t =1.94	t =−3.15	t =−3.34	
	t test	P =0.057	P =0.003	P =0.002	
Adaptability	Comparison	26.7 (4.5)	22.3(4.8)	23.5(5.4)	F = 13.54
	Intervention	24.7 (5.2)	27.3(4.8)	27.3(5.8)	P <0.001
	Independent-sample	t =1.51	t =−3.77	t =−2.43	
	t test	P =0.136	P =0.000	P =0.018	
Integrating illness into life	Comparison	19.8 (2.8)	17.3(3.3)	17.2(3.8)	F = 9.62
	Intervention	17.9(3.7)	19.8 (3.6)	19.5(3.7)	P <0.001
	Independent-sample	t =2.17	t =−2.6	t =−2.29	
	t test	P =0.035	P =0.012	P =0.026	
Sticking to treatment	Comparison	15.1 (2.9)	13.7(2.7)	13.3(2.8)	F = 5.85
	Intervention	14.2(3.4)	15.8(4)	15.8(3.2)	P = 0.004
	Independent-sample	t =1.01	t =−2.3	t =−2.93	
	t test	P =0.316	P =0.025	P =0.005	
Commitment to treatment	Comparison	14.7(3.6)	15.3(3.1)	15.9(3.2)	F =4.05
	Intervention	15.3(4.6)	19.2(3.5)	18.4(3.9)	P = 0.02
	Independent-sample	t =۰/۵۵	t =−4.33	t =−2.55	
	t test	P =0.58	P =0.000	P =0.014	
Indecisiveness for applying treatments	Comparison	10(3)	10.3(1.7)	10.4(2.4)	F = 6.52
	Intervention	10.1(2.5)	12.1(2.7)	12.2(2.4)	P <0.001
	Independent-sample	t =−0.19	t =−2.98	t =−2.63	
	t test	P =0.84	P =0.004	P =0.011	
Total treatment adherence score	Comparison	150.48(16.4)	136.19(19.8)	136.26(24)	F = 17.46
	Intervention	141.89(24)	160.26(20.9)	158.48(27.6)	P <0.001
	Independent-sample	t =1.53	t =−4.34	t =−3.15	
	t test	P =0.13	P =0.000	P =0.003	

**Figure 2.**
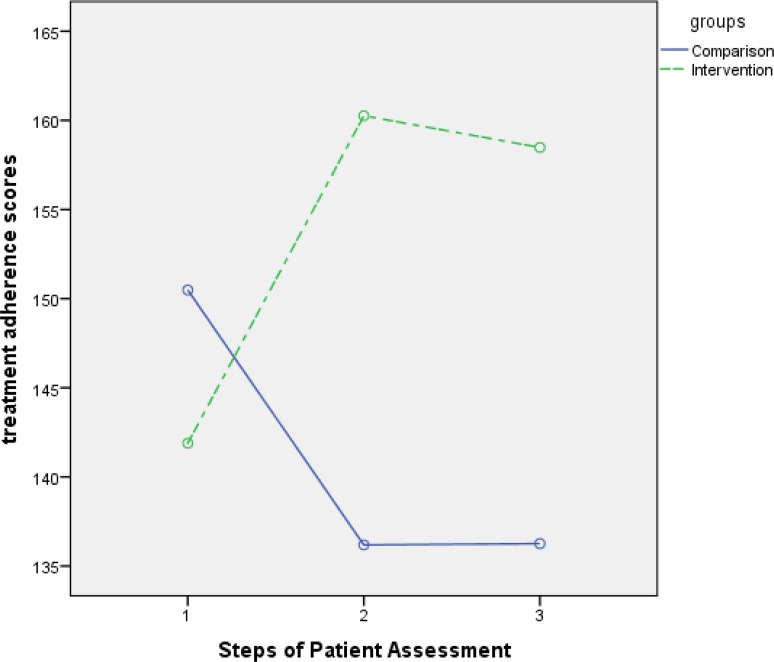
Variations of treatment adherence scores in both groups over time

However, at the second and the third time points (i.e. at the first and the second post-tests), between-group differences regarding the scores of treatment adherence and all its dimensions were statistically significant (P<0.05). The results of the repeated-measure analysis of variance also indicated significant between-group differences regarding the variations of the mean scores of treatment adherence and all its dimensions across the three measurement time points (P<0.0001)(Figure 2).

## DISCUSSION

The aim of this study was to examine the effects of MI on treatment adherence among patients with COPD. Findings showed that after the intervention, treatment adherence mean score in the MI group was significantly greater than the comparison group.

At baseline, treatment adherence in both groups was poor and there was no significant difference between the groups regarding treatment adherence mean score. Other studies also reported poor treatment adherence among patients with chronic diseases (25–27). Poor treatment adherence is associated with many different negative outcomes both for patients and healthcare systems, the most common of which include, but not limited to, disease exacerbation, short life expectancy, low quality of life, family problems, hospital over-crowdedness, healthcare providers’ heavy workload and exhaustion, and increased healthcare costs (8).

Study findings also revealed that despite routine counseling and educational services, treatment adherence in the comparison group had a downward trend while in the MI group, the trend was upward. Other studies also reported the effectiveness of MI in promoting treatment adherence among patients with psychiatric disorders (28), pneumonia (29), and hypertension (30). Similarly, Zidarn and Kolenko found that MI had significant positive effects on smoking cessation (31). Di Marco et al. also reported that MI can potentially improve the efficacy of guided self-help weight loss treatments (32). A systematic review and meta-analysis on seventeen clinical trials also reported the effectiveness of MI in promoting treatment adherence (14). The results of another meta-analysis of controlled clinical trials illustrated that when used as a primary treatment, MI produces more significant and more stable effects than when used as an independent treatment. In other words, the stability of MI effects on patients with chronic diseases largely depends on its association with other treatment strategies and strong support. Similarly, Arkowitz et al. reported that successful long-term behavioral modification necessitates the combination of MI with other treatments (33). Hence, it can be used as a primary treatment to enhance patient motivation for adhering to other treatments (34).

Despite the abundance of studies which reported the effectiveness of MI, some studies showed its ineffectiveness. For instance, Stenman et al. found that MI was ineffective in promoting treatment adherence and reducing gingival bleeding and plaque among patients with periodontal infection. These conflicting findings may be due to the fact that their MI intervention was implemented only in a single 44-minute session (35).

## CONCLUSION

MI can promote treatment adherence among patients with COPD. Of course, strong supportive, counseling, and educational interventions are needed to improve the effectiveness of MI. Future studies are recommended to assess the long-term effects of MI on treatment adherence, rehospitalization, and quality of life.
